# The Relations of the General Committee and Medical Staff of a Hospital

**Published:** 1899-01-21

**Authors:** 


### The Relations of the General Committee and Medical Staff of a Hospital.

There are few things that are more important for the well-being of a hospital than a loyal and harmonious co-operation between the medical staff and the managing committee. Whatever the older hospitals may have been in their original inception, whether they were hospices, places of refuge, lazar houses, or mere almshouses for the aged and infirm, they are now essentially medical institutions, devoted not to the mere reception and sustenance of the sick, but to their cure. Hence the preponderating importance of the medical element in all that regards hospital management. Some form then of division of labour is required, according to which he lay element shall undertake the general management of the institution, and the medical element shall take under its exclusive care all that has to do with the treatment of the patients. It must be understood that the relation between the lay and the medical elements is that of co-operation in good works, of co-partnership in a concern in which both are equally interested, and that there is no question of either being subordinate to he other. The problem is how to get arranged on a just and equitable basis the relations between the lay committees and the medical staff. Now there are several means by which this end has been sought to be attained. First, we have the rough and ready method of giving all the members of the staff a seat on the board. This at first sight seems a fair and proper recognition of the co-partnership of the medical and lay elements, and it obtains at many hospitals, notably in the provinces. But it does not work well. The personnel of committees is constantly changing; in fact, in many places it is a rule that one-third of the committee of management shall retire annually. But the staff is a fairly permanent body, and thus, if its members take an active interest in the hospital, they are able to obtain too preponderating an influence. If, on the other hand, they make it a practice not to attend meetings, but when they are interested in the subject under discussion come up in a body and outvote the lay members, serious disagreements are apt to arise, and a strong feeling of antagonism is set up between the two parties on the board. Sometimes this difficulty has been got over by arranging for the staff to elect every year two members to represent them on the general committee, or, as is done sometimes, the two senior members of the staff are made *ex-officio* members of the committee. This plan, however, has the great disadvantages that the committee are deprived of the advantage of obtaining the collective opinion of the staff on any subject under discussion, while, so far as the staff are concerned, if they wish to bring any matter before the committee they can only do so through representatives who may personally be hostile to the suggestions to be made. The result is that not only is great friction set up between the medical and the lay committees, but differences are apt to arise among the members of the staff in regard to the action of their representatives on the committee.

What, then, is the ideal plan? Clearly that the medical staff should be constituted a medical committee to which all matters of a medical character should be referred, including, of course, all that has to do with the the treatment of the patients, the regulation of the students, and the appointment of resident medical officers, and at least a large say in the appointments to the honorary posts also. The latter is a matter of supreme importance, especially at hospitals which have medical schools. We do not say that in all such affairs the decision should be absolutely handed over to the medical committee, for wherever there is co-partnership there is joint responsibility, so that, even in so entirely medical an affair as the choice of an honorary medical officer, it is probably well that the final appointment should come from the managing committee. But the medical committee should select, and the selection of the medical staff should not be lightly set aside. It must not be forgotten that the division of the committees into lay and medical is a mere division of the work according to the capacities of the workers, and involves no question of superiority. It does, however, involve a willingness on the part of each to accept the decisions of the other in its own department, and under such circumstances we cannot but feel that a refusal of the general committee to accept the recommendation of the medical committee, even though it be but a recommendation, would be a matter of great gravity, a practical repudiation of the agreement between them.

